# High Prevalence of Supplement Intake with a Concomitant Low Information Quality among Swiss Fitness Center Users

**DOI:** 10.3390/nu12092595

**Published:** 2020-08-26

**Authors:** Samuel Mettler, Joëlle Vera Bosshard, Dino Häring, Gareth Morgan

**Affiliations:** 1 Department of Health, Bern University of Applied Sciences, 3008 Bern, Switzerland; joelle.bosshard@gmx.ch (J.V.B.); dino.haering@hotmail.com (D.H.); 2 Department of Elite Sport, Swiss Federal Office of Sport, 2532 Magglingen, Switzerland; Gareth.Morgan@baspo.admin.ch

**Keywords:** supplements, information source, fitness athletes, exercise, consumer behavior, risk behavior, risk perception

## Abstract

Background: The aim of this study was to screen the prevalence of supplement use in Swiss fitness center users and what information sources they consulted. Methods: Customers of 10 fitness centers were screened with a quantitative questionnaire. Results: Eighty two percent of the 417 fitness center users consumed at least one supplement per week. Supplement intake correlated with training frequency (rs = 0.253, *p* < 0.001). The most prevalent products were protein supplements (used by 49% of the study population), magnesium (34%), and multi-micronutrient supplements (31%). The average number of supplement servings per week among consumers was 17.1 (SD: 16.1, median: 11.0) and the average number of different products used was 6.9 (SD: 4.4, median: 6.0). The most frequently used information sources were the coach/trainer (28%), the website of the supplement seller (26%), and training peers (24%). Thirty seven percent were informed or informed themselves about potential risks associated with the supplement used. The leading reasons for selecting the information source were the desire for scientific-based information followed by the education level of the informing person. Conclusions: A high prevalence of supplement intake among Swiss fitness center users was associated with a low level of information quality and a low prevalence of risk information. A discrepancy between a desire for high quality evidence-based information and a contrasting behavior was detected.

## 1. Introduction

Athletes are generally advised to follow a balanced diet adapted to the specific requirements of their individual sport [1,2]. An increase in training volume requires an appropriate change in energy and nutrient intake [3]. Additionally, many athletes add various supplements to their diet for a variety of reasons [4,5]. Health-related reasons for taking supplements may apply to the general as well as to the athletic population. Additional factors, e.g., related to performance or training adaptation, may be present among athletes. Therefore, it is not surprising to find a higher prevalence of supplement use among athletes compared to the general population [4,6].

There are different definitions for supplements used in sport. Sports supplements are often categorized into sports food (i.e., products delivering macronutrients and energy), performance supplements (supplements which directly or indirectly influence performance), and medical supplements (to prevent or treat nutritional deficiencies) [7].

Even potentially useful supplements may cause negative health or performance outcomes when used inappropriately [7]. Furthermore, there are many supplements on the market which lack evidence for any potentially useful health or performance benefit [8]. The regulations related to dietary supplements differ significantly across countries and many supplements on the international market fail to meet expected safety and efficiency standards [5,7,8,9,10,11]. Supplements may be contaminated with a large array of substances, including non-declared anabolic steroids, stimulants or heavy metals [8,10,11,12]. Unsubstantiated claims made by manufacturers may misguide athletes and expose them to harmful health effects [7,8,11].

Screening studies from around the world indicate the prevalence of supplement use among different athlete populations to be variable [4]. Limited data are available for central Europe and in particular the German speaking area, i.e., Germany, Switzerland, and Austria [4]. Representative general population data from Germany indicate that 28% use dietary supplements [13], with a higher prevalence among women (31%) compared to men (24%). A comparable prevalence (26%) was reported from a cohort in Switzerland [6]. Other countries such as the US report higher prevalence rates compared to the Swiss or German data [14], suggesting variability in the prevalence of supplement use across countries. 

Surveys generally indicate an increasing prevalence of supplement intake with age and in athletes compared to the general population [4,6,10,13]. Among athletes, there is often an increased prevalence with increasing training volume and higher performance level, i.e., elite vs. sub-elite or recreational athletes [4,5,15,16]. Braun et al. have published prevalence data among young German elite athletes [16]. There are no supplementation data available from any kind of athletic or fitness populations in Switzerland. The sports community is a diverse group including recreational gym-goers to international-level elite athletes with likely diverse supplementation behaviors. Recreational health and fitness center users are probably one of the largest groups. About 16% of the Swiss Population is a member of a private fitness center and holds a subscription for regular training sessions [17]. The goal of the present study was to collect data about the prevalence of supplement use in fitness center users in Switzerland. In addition, motivation for supplement intake and information sources were examined.

## 2. Materials and Methods

### 2.1. Recruitment and Ethics

Ten fitness centers in the Bern region of Switzerland participated in this study (three being branches of the same company) and agreed to have their customers surveyed for the purpose of this study. The participating centers included low budget as well as medium to high priced “premium” centers and represented a broad range of customers. Data collection was performed in February 2019 with a quantitative questionnaire. The study sample was defined as all customers entering the fitness centers during screening time. Screenings were held according to study team access to the different fitness centers. In four fitness centers screenings were held over one day (8 h per day). In the other fitness centers screening occurred over two evenings (3 h per evening). Different weekdays were selected. Customers entering the fitness centers were informed both orally and in writing about the study goal. They were also notified that participation was voluntary, that placing the anonymous questionnaire in the anonymous collecting box was considered as informed consent to participate in the survey, and that any question may be skipped or incomplete questionnaire returned. The collecting box was opened by the research team at the end of each screening day. All data were collected anonymously. The study was carried out in accordance with the Declaration of Helsinki.

### 2.2. Inclusion and Exclusion Criteria

Inclusion criteria were: All customers who entered the fitness centers during the time window of the screening in the respective fitness center and who were at least 18 years old.

Exclusion criteria were: Age under 18 years. Empty questionnaires and questionnaires of subjects under 18 years of age were excluded. Partially completed questionnaires were included, provided the second section (supplement intake) was completed. If the supplement intake section was not completed the questionnaire was excluded.

Language restriction: The questionnaire was available in German only, representing the official area language and the corresponding language in the participating fitness centers. A very small minority of the customers might have been unable to participate due to language restrictions.

### 2.3. Questionnaire

The quantitative questionnaire was divided into three sections on a single double-sided A4 sheet of paper. In the first section, categorical variables were screened, including gender, age group, training frequency per week (Table 1), and main training goals (i.e., support health, weight loss, body shaping, bodybuilding, improve strength, improve endurance, improve sport specific performance, other reasons). In the second section, the supplement use was screened by listing 25 predefined substances and product classes (Table 2). Spare lines were available to list “further” products not included in the questionnaire. For each product, intake frequency had to be indicated. The available categories were “daily”, “several times per week”, “once a week”, “less than once a week”, “never”, and “don’t know”. In the last section, subjects were asked about their motivation to take the supplements, where they informed themselves about the supplements used, whether they were informed about potential side effects or risks by their information source (yes or no) and what they considered to be important criteria when selecting the particular information source(s). For these questions, a limited set of predefined answers was presented (see the corresponding tables and figures in the result section) including an open answer to insert further individual responses. For some predefined answers, a dependent open question was added. For example, one answer for the question about the used information sources was “I took courses on the subject “. In this case, the participants were asked what kind of course they completed. In order to limit the required time to fill in the questionnaire and to optimize response rate as much as possible, the questionnaire and the predefined answers were intentionally limited to one single double-sided page. Multiple answers were possible.

The questionnaire inquired about current supplement intake, defined as the last four weeks. Subjects were classified as consumers if they consumed at least one supplement at least once a week. Participants with a more sporadic intake, i.e., “less than once a week”, “never” or “don’t know” were classified as non-consumers. A pretest of the questionnaire was performed with 15 fitness center users to test for applicability and understandability of the questionnaire. Reliability was tested by asking the same subjects a week later to fill in the same questionnaire again. These questionnaires were not included in the analysis.

### 2.4. Statistics

Descriptive data analysis was performed with Microsoft Excel 365 ProPlus (2019). IBM SPSS Statistics 26.0.0.0 was used to perform Chi-Square and Spearman rank correlation tests. A Chi-Square test was used to identify differences between supplement intake prevalence between genders and age groups. For supplement intake prevalence among age groups, logistical regression analysis was used for post-hoc analysis in the case of a significant Chi-Square test. Bonferroni adjustment was used to account for multiple comparisons. To estimate the average number of supplement servings per week, the intake frequency of all products was summed up. For this reason, the assigned servings per week were seven for “daily intake”, 3.5 for “several times per week”, one for “once a week”, 0.5 for “less than once a week”, and zero for the further ratings. 

In order to compare our study population to the total client base of the screened fitness centers we compared the average age of our study sample with the corresponding population mean. Due to data privacy reasons the average age of their total client base was the only information we got from all fitness centers. Average age for our study population was calculated as average age of the selected age category or as 68 y for the category >60 y of age. Otherwise, age was handled as a categorical variable.

In order to calculate the response rate, we tracked the number of all customers entering the fitness center during the screening phase. The response rate was calculated by dividing the number of returned questionnaires (without empty or excluded questionnaires) by the numbers of counted customers entering the fitness center during the screening phase. 

## 3. Results

### 3.1. Subjects

In total, 417 fitness center users were included in the analysis, representing men (49%), women, and different age groups. Training frequencies ranged from one to two training sessions a week (21%) to daily sessions (6%, Table 1). The average response rate was 59%. Average client age in the respective fitness center databases (38 y) was comparable to the average age of the study sample subjects (37 y).

### 3.2. Supplement Intake

Overall, 82% of all fitness center users consumed at least one supplement per week and were thus classified as consumers (Table 2 and Table 3). The most prevalent products used were protein powders and drinks (used by 43% of the study population), magnesium (34%), and multi-micronutrient supplements (31%). If solid protein bars and liquid protein products (protein powder and drinks) are combined, 49% of all subjects consumed at least one of these protein supplements. Protein supplements were significantly (*p* < 0.001) more prevalent among men (62%) than women (35%). The average number of supplement servings per week among consumers was 17.1 (SD: 16.1, median: 11.0) and the average number of different products used was 6.9 (SD: 4.4, median: 6.0). Among consumers, 31%, 17%, and 10% of the fitness center users consumed more than 20, 30, or 40 supplement servings per week, including one subject taking 100 supplements per week while training less than once a week.

**Figure 1:**
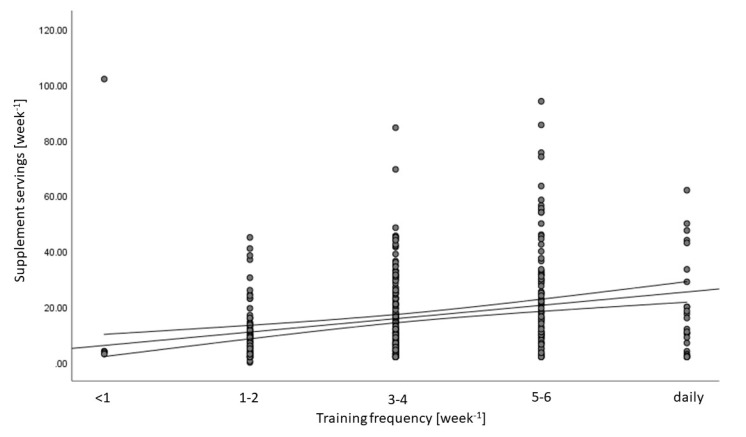
Supplement intake vs. training frequency. The scatter density plot and spearman rank correlation (mean and 95% confidence interval) of supplement servings per week vs. training frequency give an r_s_ = 0.253 (*p* < 0.001).

The number of supplement servings per week showed a weak positive correlation (*p* < 0.001) with training frequency (Figure 1). 

### 3.3. Training Goals

**Figure 2:**
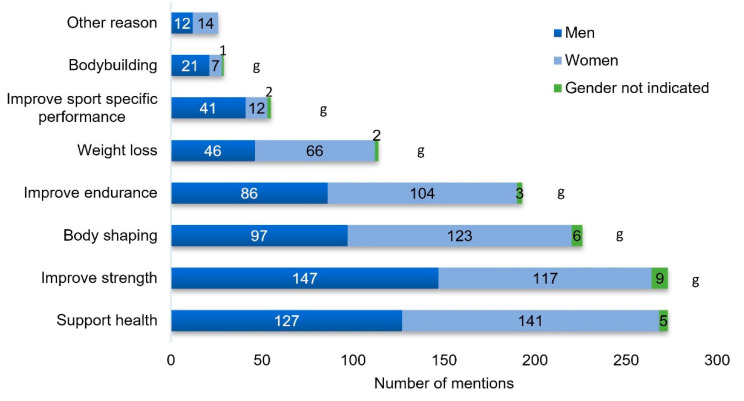
Training goals. g: Indicates significant gender effect (*p* < 0.05). Multiple answers were possible.

The three most important training goals of the participating fitness center users were improving health, improving strength, and body shaping (Figure 2). Analysis by age showed obvious age effects for two training goals. Improving health was named by 59%, 60%, 75%, and 89% as a training goal among the four age groups beginning from the youngest to the oldest group. On the other hand, body shaping was named by 69%, 55%, 41%, and 17% among the four age groups. 

### 3.4. Reasons for Supplement Intake

**Figure 3:**
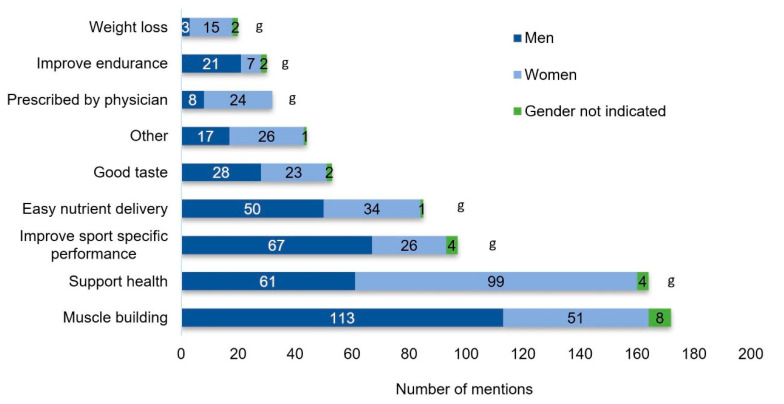
Reasons for supplement use. g: Indicates significant gender effect (*p* < 0.05). Multiple answers were possible.

The three most important reasons to take supplements were muscle building (49%), improving health (47%), and improving sport-specific performance (28%) (Figure 3). 

### 3.5. Information Sources

**Figure 4:**
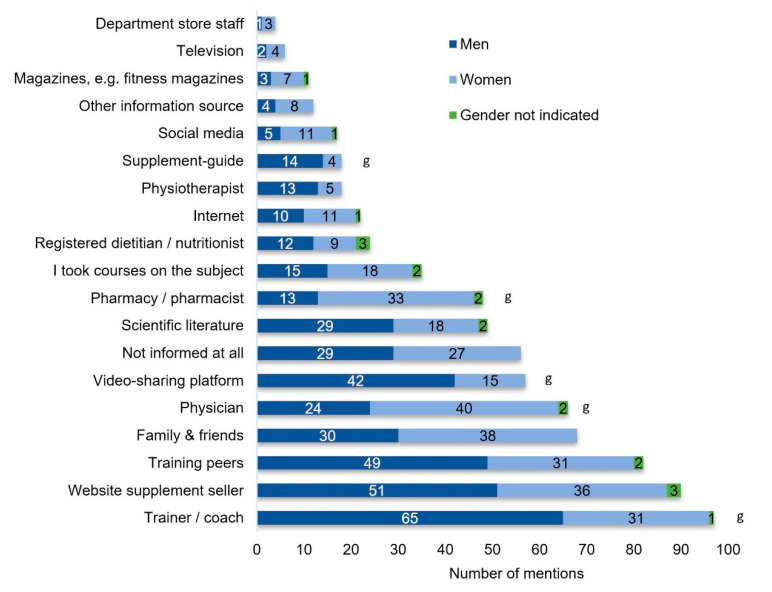
Indicated relevant information sources about supplements. Multiple answers were possible. g: Indicates significant gender effect (*p* < 0.05).

Regarding used information sources, the top three were the coach/trainer (28%), the website of the supplement seller (26%), and training peers (24%) (Figure 4). On average 2.2 information sources were indicated by the fitness center users. The question regarding information about risks and side effects was answered by 315 subjects. Only 117 (37%) of these had received information regarding potential risks and side effects of the consumed supplements. The three leading information sources for risks and side effects were the internet, physicians, and coaches/trainers. When subjects answered that they were the source of information themselves, only two of them had an accredited (para)medical education (i.e., registered dietitian or physician). The most prevalent answers were different types of fitness instructor courses (13) and sport studies (5).

**Figure 5:**
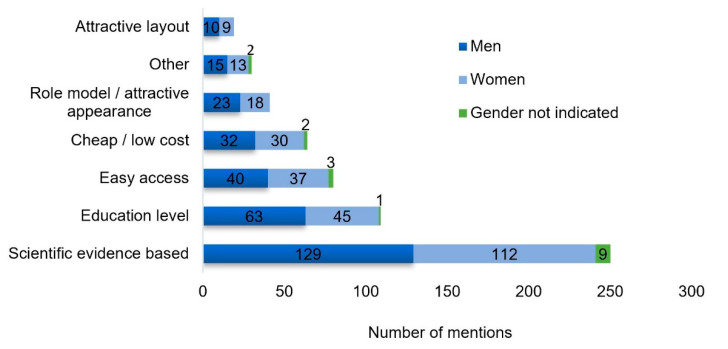
Reasons for information source selection. No significant gender effects (*p* < 0.05).

Leading reasons for selecting the specific information sources were the desire for science-based information, followed by the “education level of the informing person”, and “easy access” to the information (Figure 5).

Among all subjects, 37% were informed or informed themselves about potential risks associated with the supplement used. There was no significant gender (*p* = 0.36) or age (*p* = 0.52) effect regarding information obtained about risks. 

## 4. Discussion

### 4.1. Supplement Intake

The aim of this study was to gain insight into the prevalence of supplement intake and used information sources among recreational fitness center users in Switzerland. Overall, 82% of the fitness center users consumed supplements. This prevalence is higher compared to a general population sample in Switzerland, where only 26% used supplements [6]. It is also higher in comparison with fitness athlete populations from other countries and cultural regions [4,18,19,20], and even compared to reports from some elite athlete populations [4,16]. The definition of supplements and supplement use differs across studies, i.e., intake prevalence depends on whether or not subjects are asked about their current intake, which is often defined as the last four weeks as in the present study [4,16]. When subjects are asked for longer timeframes, the prevalence usually increases [16,21]. Prevalence might be influenced by the sport and training goals of the specific population being screened [16], e.g., among body builders, prevalence rates of 100% were reported [22]. However, the few subjects indicating body building as their training goal in our study cannot explain our results. The relatively high purchasing power in Switzerland might also contribute to the comparatively high prevalence of supplement use, or is at least not a barrier. 

The median number of weekly supplement servings indicated that 50% consumed more than 11 servings per week, while there was a subset of consumers with quite substantial supplement use, i.e., 10% took >40 supplements per week. The number of supplement servings per day or week has usually not been reported in other studies. This might be explained by the fact that other studies also used categorical variables to assess supplement intake frequency. To calculate a summed-up number of supplement servings, assumptions had to be made by allocating numbers to the categories. This is sometimes quite straightforward as for the categories “once a week” or “daily”, but may be afflicted with errors. Even for the seemingly straight forward category “daily”: If one serving per day is the assumption, this might be an underestimate as there was no category for a more frequent intake. Somebody taking several servings a day still could only mark “daily” intake. Hence, the calculated number of servings could likely be underestimated. Nevertheless, the number may represent an educated guess of the magnitude of supplement intake, which seems to be even more relevant than the mere prevalence of supplements in use. Future studies should focus more on this parameter to obtain more data about the scale of supplement intake and not only about the prevalence of supplement users, no matter the extent of use. Our data indicate that a significant part of the supplement consumers is supplementing on a large scale, i.e., 31% use at least 20 supplement servings per week and 10% use 40 or more supplement servings per week. 

Protein supplements represented the most prevalent products, which is in line with other supplement screening studies [19,23,24]. In line with others [4], we reported a more prevalent protein intake among men compared to women. This correlates with the more prevalent training goal of muscle and strength gain among men. We detected more prevalent protein and creatine supplementation among younger compared to older fitness center users, whereas the opposite was found for vitamin supplementation. Other authors made similar observations [23] and it might reflect the different training goals among age groups, i.e., more focus on strength and muscles among the younger and more focus on health among the older age groups. 

Comparable to other studies [4], we detected a positive correlation between training frequency and supplement usage. Nevertheless, the correlation is weak and is definitely not a predictor at the individual level. The subject with the highest supplement intake of 100 weekly servings was training only once a week. 

### 4.2. Information Sources

Although athletes are generally not advised to consult friends, training peers or coaches as primary information sources about supplements [25], the present study population declared trainer, coaches, websites of supplement sellers, training peers, and family members or friends as the leading information sources. Trainers’ and coaches’ nutrition knowledge may be inadequate and peer experience is also unlikely to provide an evidence-based and individualized supplement regime [26,27,28]. Unfounded endorsement or encouragement of supplement use by influential individuals in the athlete’s circle such as coaches and training peers may also explain the relatively high supplement use in the present study [8]. Unfiltered information from shared online videos and websites is obviously an influential source as well. Physicians and registered dietitians do not seem to be a predominant information source, which is in line with other studies [24]. 

It is very plausible that this behavior increases safety risks for athletes [25], and that these information sources are insufficiently qualified or biased. Most supplements on the market are of limited value for consumers [8] and it is highly unlikely that a supplement seller will post any information that might curtail sales. Mislabeling and contamination of dietary supplements with undeclared substances, i.e., anabolic steroids, pharmaceuticals, heavy metals or stimulants with concomitant health risks represents a widespread problem in the supplement market [5,8].

We detected an obvious discrepancy between the study participants’ selected information sources and their expectations regarding the information quality. The desire for evidenced-based information was in sharp contrast to the obviously nutritionally uneducated persons or entities chosen as primary information sources. 

This discrepancy may have two distinct reasons. Either the subjects are aware of the mismatch, but they are unwilling to close the gap, because the chosen resources are easily available and mostly free of charge (internet information, colleagues or the fitness trainer provides nutrition and supplement recommendations as a side activity). Alternatively, the subjects are not aware of the mismatch and are therefore not able to bring their quality expectations in line with their behavior. This would indicate that people are largely unable to distinguish evidence-based information and educated professionals from nutritionally insufficiently educated information sources such as fitness instructors, peers or supplier websites. This hypothesis might be supported by the observation that physiotherapists are used nearly as often as sources of information as dietitians or nutritionists, although there is obviously a dramatic nutritional education difference between these two paramedical professions. 

Those who designated themselves as being educated indicated, with few exceptions, courses that would not qualify somebody to provide substantiated nutrition and supplementation advice. On the one hand these persons might overestimate their own degree of knowledge and power of judgment. At the same time, these same individuals often serve as primary information sources for athletes (i.e., fitness trainers working in the fitness centers); customers obviously accept them as valuable information source. 

The Dunning-Kruger effect refers to a cognitive bias in which individuals with a low level of knowledge in a particular subject mistakenly assess their knowledge or ability as greater than it is [29]. This effect may likely be attributed to the subjects who designated themselves as being educated in the field, though the vast majority of them had no more than a few hours of nutrition training. In addition, it may be that personal experience with a product is mistaken for knowledge. This may relate to family members, friends, and training peers being accepted as trusted information sources. 

We may also speculate about so-called authority bias, the tendency to attribute greater accuracy to the opinion of an authority figure, unrelated to its content [30]. This may explain why fitness instructors or physiotherapists are so often considered comparable to qualified dietitians. 

It becomes clear that taken together (family members, friends, and training peers), the predominant group of influential individuals regarding dietary supplementation is completely unqualified medically. In this case, it is likely that the trust that has been established in these individuals is mistaken for professional expertise. 

Consequently, this study indicates significant demand for identifying reliable information sources amongst our fitness center users. In contrast to this study, a recent study with high level athletes affiliated with an Australian state-based sports institute indicated that nutritional and medical staff were among the most influential sources of information regarding supplement use, while friends or supplement supplier information were rated as least influential [31]. This might indicate that athletes in close contact with professional support might have and accept more influence from professional support and might also be able to better differentiate between evidence-based professional advice and lay information. Another reason might be the athlete’s performance level. An Australian study reported that non-elite athletes are more likely to choose the internet and less likely to choose a dietitian or nutritionist as primary information sources compared to elite athletes [32]. Overall, the internet (47%), trainers (42%), and family/friends (37%) were the leading “top 3” information sources amongst a range of elite and non-elite Australian athletes. However, dietitians (41%) and nutritionists (37%) were also frequently named in the ‘top 3’ information sources [32]. This is different to our gym-going health and fitness athletes where dietitians and nutritionists together were named by just 5.7%. As the athletic level of our gym-goers was very likely even lower than the non-elite Australian athletes, the observation that the level of athleticism might be one possible predictor for poor information source choice, would be supported by our data. 

The low percentage (37%) of the study population who informed themselves or were informed about risk factors associated with dietary supplement intake may also be a consequence of the largely uneducated or unqualified information sources considered. Importantly, we only asked whether or not information about the risks had been given. We did not evaluate whether the information was appropriate. If the information source about supplement intake risk was indicated by the subjects, sources such as the Internet, YouTube, and fitness instructors were again frequently named. Consequently, it is likely that many of the 37% were informed in an unfounded manner regarding risk factors. Therewith, this study clearly indicates insufficient safety knowledge among these product users and a need for broad information dissemination about potential risks associated with dietary supplement use. 

Although the questionnaire was not designed to evaluate the appropriateness of supplement use, there were several indications that supplements were used inappropriately. On the one hand, ineffective supplements such as fat burning products were used. At the same time, potentially effective supplements such as creatine or beta-alanine were used only once a week, which cannot be effective [9], indicating random supplement use. This may be a further consequence of insufficient knowledge among the study population. Moreover, excessive use of supplements such as 100 supplement servings per week is rather unlikely to reflect a targeted supplement use.

### 4.3. Limitations

Certainly, this study has some limitations. Firstly, we could only screen a limited number of fitness centers. Secondly, the overall response rate of 59% did not exclude a certain selection bias. Nevertheless, we managed to include more subjects than many other studies [4] and the available indicator (average age) does not speak against the hypothesis that the study population might more or less represent the population of the screened fitness centers. Furthermore, the questionnaire was intentionally reduced to one two-sided A4 paper in order to keep it simple and short and with the intention to focus on return rate rather than on comprehensiveness. Therefore, the number of questions and the number of predefined answers per question were limited. This might have influenced the answers to some degree. 

## 5. Conclusions

In conclusion, this study indicates a high prevalence of supplement use among Swiss fitness center users. The high use was associated with a low level of information quality. We detected a striking discrepancy between an obvious desire for high quality evidence-based information and a blatant contrasting behavior. The predominant information sources, including trainers, coaches, websites of supplement sellers, training peers, friends, and family members were clearly inappropriately educated to provide evidence-based and unbiased information. This was underlined by the limited knowledge about potential risks associated with dietary supplement use among supplement users. This study clearly indicates a significant demand for empowering the target population in identifying reliable sources of evidence-based information as well as in understanding potential risks associated with dietary supplement use. 

## Figures and Tables

**Table 1:** Subject characteristics as percent and frequency (*n*) of the study population.

	%	*n*
Gender		
Male	49	205
Female	48	200
Not given	3	12
Age group [y]		
18–30	47	197
31–45	23	97
46–60	15	61
>60	11	47
Not given	4	15
Training		
Frequency		
[week^−1^]		
<1	1	5
1–2	21	89
3–4	50	210
5–6	21	86
Daily or more	6	25
Not given	1	2

**Table 2:** Supplement intake by gender as percent and frequency (*n*).

	Total (*n* = 417) ^2^ % (*n*)	Men (*n* = 205) % (*n*)	Women (*n* = 200) % (*n*)	*p* (Gender)
Consumers ^1^	82.0 (332)	84.4 (173)	80.0 (159)	0.201
Protein powders and drinks	43.2 (180)	54.6 (112)	30.0 (60)	<0.001
Magnesium	33.7 (141)	30.2 (62)	36.5 (73)	0.182
Multivitamin and multimineral	31.4 (131)	32.2 (66)	29.5 (59)	0.557
Vitamin D	24.0 (100)	21.5 (44)	25.5 (51)	0.338
Sports drinks	22.3 (93)	30.7 (63)	13.0 (26)	<0.001
Vitamin C	20.1 (84)	23.4 (48)	16.5 (32)	0.061
Recovery products	19.2 (80)	24.4 (50)	13.5 (27)	0.005
Protein bars	18.0 (73)	21.0 (43)	15.0 (30)	0.118
Amino acids (e.g., BCAA, glutamine)	17.3 (72)	22.0 (45)	12.0 (24)	0.008
Energy drinks	14.4 (60)	14.6 (30)	14.5 (29)	0.969
Caffeine (without coffee)	13.2 (55)	16.1 (33)	9.5 (19)	0.047
Creatine	12.9 (54)	22.4 (46)	3.5 (7)	<0.001
Iron	11.0 (46)	10.2 (17)	14.0 (28)	0.068
Energy bars	8.6 (36)	10.7 (22)	6.5 (13)	0.130
Plant extracts	7.9 (33)	10.2 (17)	7.5 (15)	0.767
L-Carnitine	7.4 (31)	9.8 (20)	5.0 (10)	0.068
Beta-Alanine	4.1 (17)	5.4 (11)	1.5 (3)	0.033
Alkalizing mineral products	3.4 (14)	2.9 (6)	3.5 (7)	0.744
Probiotics	3.1 (13)	2.4 (5)	3.5 (7)	0.529
Carbohydrate gels	2.2 (9)	3.4 (7)	0.5 (2)	0.099
Nitrate/Beetroot juice	1.9 (8)	1.5 (3)	2.5 (5)	0.454
Fat-Burn Products	1.7 (7)	2.0 (4)	1.5 (3)	0.728
Bicarbonate/Citrate	0.7 (3)	0.5 (1)	0.5 (1)	0.968
HMB	0.5 (2)	0.5 (1)	0.0 (0)	0.323
Steroids	0.2 (1)	0.0 (0)	0.0 (0)	-
Other	4.1 (17)	4.4 (9)	4.0 (8)	0.845

Product/substances are listed in order of their intake prevalence. ^1^ Consumed at least one supplement at least once per week. ^2^ Including men, women, and subjects with non-declared gender.

**Table 3:** Supplement intake prevalence by age group.

	18–30 (*n* = 197) % (*n*)	31–45 (*n* = 97) % (*n*)	46–60 (*n* = 61) % (*n*)	>60 (*n* = 47) % (*n*)	*p* (Age)
Consumers ^1^	87.3 (172) ^d^	79.4 (77)	82.0 (50)	66.0 (31) ^a^	0.006
Protein powders and drinks	55.8 (110) ^c,d^	45.4 (44) ^c,d^	19.6(12) ^a,b^	14.9 (7) ^a,b^	<0.001
Magnesium	35.0 (69)	29.9 (29)	37.7 (23)	31.9 (15)	0.733
Multivitamin and multimineral	33.5 (66)	27.8 (27)	31.1 (19)	31.9 (15)	0.808
Vitamin D	23.9 (47)	21.6 (21)	21.3 (13)	25.5 (12)	0.931
Sports drinks	24.4 (48)	18.6 (18)	32.8 (20) ^d^	6.4 (3) ^c^	0.007
Vitamin C	20.8 (41)	18.6 (18)	19.7 (12)	17.0 (8)	0.930
Recovery products	27.4 (54) ^d^	15.5 (15)	11.5 (7)	4.3 (2) ^a^	<0.001
Protein bars	18.8 (37)	18.5 (18)	24.5 (15)	8.5 (4)	0.200
Amino acids (e.g., BCAA, glutamine)	24.8 (49) ^c^	14.4 (14)	6.5 (4) ^a^	8.5 (4)	0.001
Energy drinks	22.8 (45) ^b,c^	8.2 (8) ^a^	6.5 (4) ^a^	2.1 (1)	<0.001
Caffeine (without coffee)	19.8 (39)	9.3 (9)	4.9 (3)	4.3 (2)	0.001 ^£^
Creatine	19.8 (39) ^c^	11.3 (11)	3.3 (2) ^a^	4.3 (2)	0.001
Iron	15.2 (30)	9.3 (9)	8.2 (5)	2.1 (1)	0.046 ^$^
Energy bars	8.6 (17)	7.2 (7)	13.1 (8)	6.4 (3)	0.555
Plant extracts	10.7 (21)	6.1 (6)	6.6 (4)	4.3 (2)	0.349
L-Carnitine	8.1 (16)	6.2 (6)	11.5 (7)	4.3 (2)	0.500
Beta-Alanine	5.1 (10)	4.1 (4)	1.6 (1)	2.1 (1)	0.589
Alkalizing mineral products	2.0 (4)	6.2 (6)	1.6 (1)	4.3 (2)	0.234
Probiotics	3.0 (6)	3.1 (3)	4.9 (3)	2.1 (1)	0.858
Carbohydrate gels	2.0 (4)	2.1 (2)	3.3 (2)	0.0 (0)	0.687
Nitrate/Beetroot juice	1.0 (2)	1.0 (1)	1.6 (1)	6.4 (3)	0.078
Fat-Burn Products	3.0 (6)	1.0 (1)	0.0 (0)	0.0 (0)	0.245
Bicarbonate/Citrate	0.0 (0)	2.1 (2)	1.6 (1)	0.0 (0)	0.190
HMB	0.5 (1)	1.0 (1)	0.0 (0)	0.0 (0)	0.778
Steroids	0.0 (0)	1.0 (1)	0.0 (0)	0.0 (0)	0.369
Other	5.5 (11)	1.0 (1)	3.3 (2)	2.1 (1)	0.238

For readability, product/substances are sorted according to Table 2; ^1^ Consumed at least one supplement at least once per week; ^$^ After a Bonferroni adjustment no significant differences remained. Without a Bonferroni adjustment the age group 18–30 differed from the age group >60 (unadjusted *p* = 0.040); ^£^ After a Bonferroni adjustment no significant differences remained. Without a Bonferroni adjustment the age group 18–30 differed from all other age groups: Unadjusted *p* = 0.025 (against age group 30–45), *p* = 0.012 (45–60), *p* = 0.021 (>60); ^a^ significantly different from age group 18–30; ^b^ significantly different from age group 30–45; ^c^ significantly different from age group 45–60; ^d^ significantly different from age group >60.
